# Flexible pulse-controlled fiber laser

**DOI:** 10.1038/srep09399

**Published:** 2015-03-24

**Authors:** Xueming Liu, Yudong Cui

**Affiliations:** 1 State Key Laboratory of Transient Optics and Photonics, Xi'an Institute of Optics and Precision Mechanics, Chinese Academy of Sciences, Xi'an 710119, China

## Abstract

Controlled flexible pulses have widespread applications in the fields of fiber telecommunication, optical sensing, metrology, and microscopy. Here, we report a compact pulse-controlled all-fiber laser by exploiting an intracavity fiber Bragg grating (FBG) system as a flexible filter. The width and wavelength of pulses can be tuned independently by vertically and horizontally translating a cantilever beam, respectively. The pulse width of the laser can be tuned flexibly and accurately from ~7 to ~150 ps by controlling the bandwidth of FBG. The wavelength of pulse can be tuned precisely with the range of >20 nm. The flexible laser is precisely controlled and insensitive to environmental perturbations. This fiber-based laser is a simple, stable, and low-cost source for various applications where the width-tunable and/or wavelength-tunable pulses are necessary.

Fiber-based lasers attract extensive attention due to their various advantages such as compactness, reliability, and high stability^1^,^2^,^3^,^4^,^5^,^6^,^7^,^8^,^9^,^10^,^11^,^12^. A variety of mode-locking techniques have been developed to make pulse lasers as versatile tools for many applications in fiber telecommunication, optical frequency comb generation, metrology, and microscopy^13^,^14^,^15^,^16^,^17^,^18^,^19^,^20^,^21^,^22^,^23^. So far, a number of saturable absorbers (e.g. the nonlinear polarization rotation^24^,^25^,^26^,^27^,^28^,^29^,^30^, nonlinear optical loop mirror^31^,^32^,^33^, semiconductor saturable absorber mirror^34^,^35^,^36^,^37^,^38^, graphene^39^,^40^,^41^,^42^,^43^, and carbon nanotube (CNT)^44^,^45^,^46^,^47^) have been proposed to implement the mode-locking operation. Among them, CNTs are particularly interesting for pulse generation because of highly environmental stability and being insensitive to the polarization of pulses evolving in the laser cavity^45^,^46^,^47^,^48^,^49^,^50^,^51^.

To control the laser property, a filter is widely employed into the laser cavity. For instance, a birefringent-plate-based filter can stabilize high-energy pulses in the all-normal-dispersion fiber lasers^52^. But its operation wavelength is fixed, as well as its spectral bandwidth is inflexible because it attributes to the thickness of birefringent plate^26^,^52^. Wavelength tuning can be realized by using an intracavity bandpass filter^46^,^53^, whereas the spectral bandwidth and the pulse width are still inflexible. Fortunately, fiber Bragg grating (FBG) is an ideal component for fiber lasers because it can provide the changeable dispersion and the tunable transmittance wavelength together with negligible nonlinearity^54^,^55^. Then, FBG offers the great flexibility for controlling the wavelength of the generated pulses. However, the pulse width of lasers is usually fixed although it can be tuned slightly by changing the pump power or adjusting the components of cavity^45^,^46^. To address this issue, we have proposed a flexible technique by means of controlling FBG.

In this article, we report a compact pulse-controlled fiber laser for the first time to our best knowledge, in which the pulse width and the pulse wavelength can be controlled precisely by adjusting FBG. The controlled scalable range of pulse width in the proposed fiber laser is accurately tunable from ~7 to ~150 ps. The wavelength of pulse is precisely tunable with the range of >20 nm. This laser is insensitive to environmental perturbations and thus is viable for various practical applications.

## Results

### Experimental setup of controlled flexible laser

**Figure 1:**
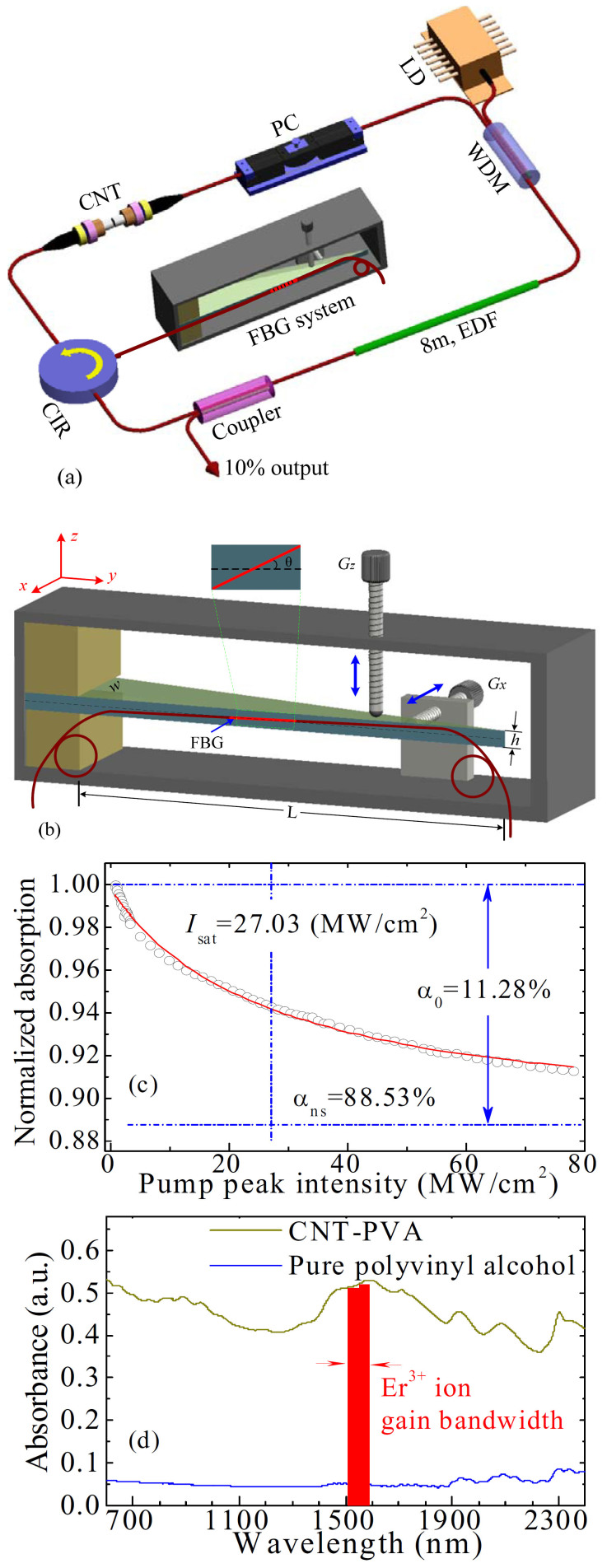
(a) Laser setup. EDF, erbium-doped fiber; WDM, wavelength-division multiplexer; PC, polarization controller; LD, laser diode; CIR, circulator; FBG, fiber Bragg grating; CNT, carbon nanotube. (b) FBG system. A uniform FBG is glued in a slanted direction onto the lateral side of a right-angled triangle cantilever beam. The flexible cantilever beam is made of polyurethane. (c) Nonlinear absorption characterization of the CNT-SA. The solid curve is fitted from the experimental data (circle symbols). (d) Absorption spectra of the pure polyvinyl alcohol (PVA) and the CNT-PVA composite. The red stripe illustrates the spectral gain region of the Er^3+^-doped fiber.

The schematic diagram of controlled flexible fiber laser is shown in Fig. 1(a). The proposed laser consists of a FBG system, a circulator (CIR), a CNT saturable absorber (SA), a polarization controller (PC), a wavelength-division multiplexer (WDM), an 8-m-long erbium-doped fiber (EDF) with 6 dB/m absorption at 980 nm, a fused coupler with 10% output ratio, and a piece of single-mode fiber (SMF). The dispersion parameters of EDF and SMF are about 11.6 and −22 ps^2^/km at 1550 nm, respectively. The total cavity length is ~21.5 m.

The key component of this laser, shown in Fig. 1(b), is a FBG system that introduces the accurately width-tunable and wavelength-tunable operations for pulse generation. A uniform FBG is glued in a slanted direction onto the lateral side of a right-angled triangle cantilever beam with length *L* = 18 cm, width *w* = 3 cm, and thickness *h* = 1.2 cm. The flexible cantilever beam is made of polyurethane with the high resistance against fatigue. The FBG is carefully attached by using the UV curable epoxy. The center of FBG lies on the neutral plane of the beam, as shown in inset of Fig. 1(b). The angle, *θ*, between the axis of the FBG and the neutral layer of the beam is about 15°.

The integrated CNT-based SA is made by sandwiching a ~2 mm^2^ sample between two fiber connectors. The fabrication procedure is shown in our previous report^45^. Figure 1(c) shows the normalized nonlinear absorption of CNT-SA, which is experimentally measured with a homemade ultrafast laser at 1550 nm. The experimental data are fitted as the solid curve of Fig. 1(c) on the basis of a simplified two-level SA model^45^. Figure 1(c) illustrates that the linear limit of saturable absorption (*α*_0_), the nonsaturable absorption (*α*_ns_), and the saturation intensity (*I*_sat_) are about 11.28%, 88.53%, and 27.03 MW/cm^2^, respectively. Figure 1(d) demonstrates the absorption spectra of the pure polyvinyl alcohol (PVA) and the CNT–PVA composite, which are measured by a spectrometer (JASCO V-570 UV-vis-NIR). It is worth noting that the tube diameter of CNTs is ranging from 0.8 to 1.3 nm here, whereas it is less than 2 nm in Ref. 45.

### Bandwidth-tunable and wavelength-tunable operations

**Figure 2:**
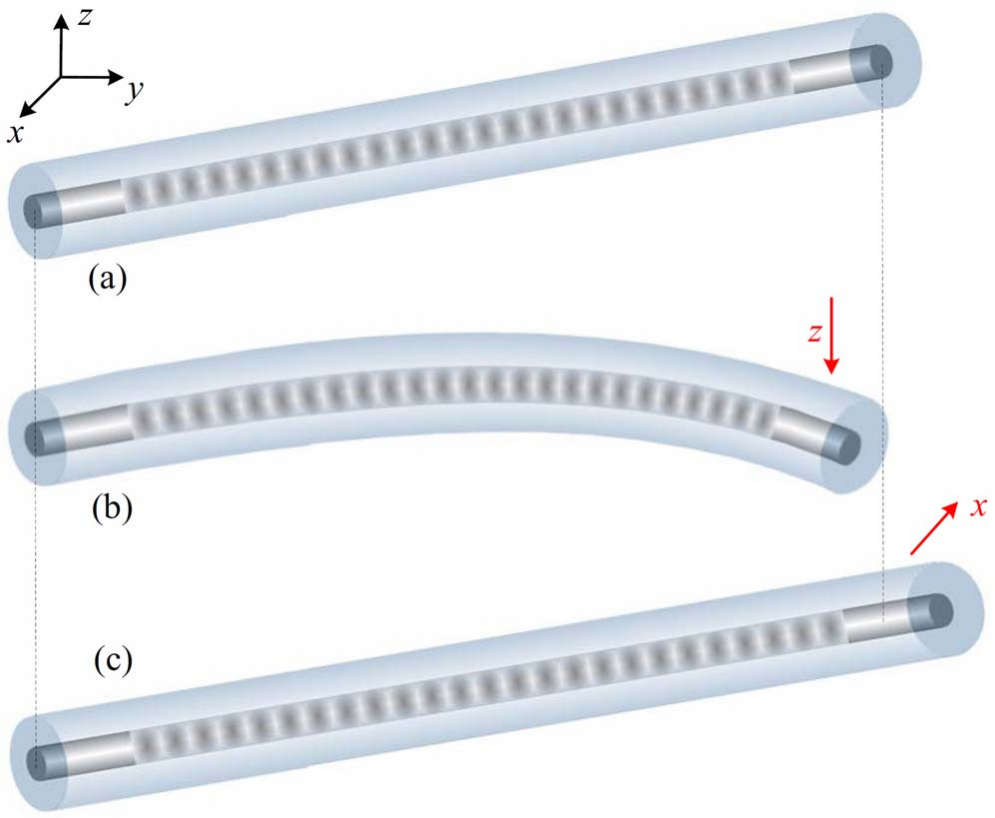
Schematic diagram of bandwidth-tunable and wavelength-tunable operations by flexibly controlling FBG. (a) Without the translation in the free state, (b) vertically translating the screw *G_z_* along the direction of *z*-axis, and (c) horizontally translating the screw *G_x_* along the direction of *x*-axis.

**Figure 3:**
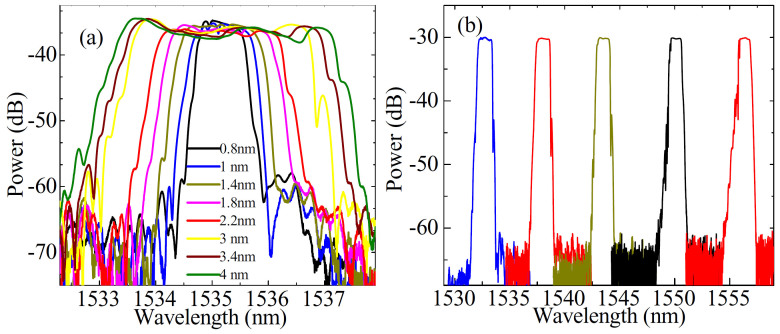
Typically reflection spectra of FBG. (a) Vertically translating the screw *G_z_* along *z*-axis. The FBG bandwidth Δ*λ_B_* is changed whereas the central wavelength approximately is fixed. Δ*λ_B_* is about 0.8, 1, 1.4, 1.8, 2.2, 3, 3.4, and 4 nm from inner to outer, respectively. (b) Horizontally translating the screw *G_x_* along *x*-axis. The central wavelength of FBG is tuned with the range of >20 nm, whereas the spectral profile changes slightly.

The beam is bent when the screw *G_z_* is translated along *z*-axis, as shown in Fig. 1(b). The half of the grating is under varying tension, whereas the other half is under varying compression. If the center of the grating is located exactly at the neutral layer of the beam, there will be no strain at the center of the grating^57^,^58^. In this case, the vertical distance shift at the FBG center is equal to zero and then the central wavelength shift Δ*λ_c_* is also equal to zero according to Eqs. (1) and (2) (see METHODS). Figure 2(b) shows the induction principle of tension and compression strain along the FBG based on the symmetrical bending. As a result, the bandwidth Δ*λ_B_* of FBG can be flexibly controlled by the tension and compression strain at each side of the FBG without the shift of the central wavelength. Figure 3(a) shows some typically experimental results of reflection spectra of the FBG with the variation of translation. The bandwidth of FBG is changed in the range from about 0.8 to 4 nm. Obviously, the central wavelength approximately remains fixed although the bandwidth-tunable operation is realized.

When the screw *G_x_* in Fig. 1(b) is translated horizontally (i.e., along the direction of *x*-axis), the variation of FBG bandwidth Δ*λ_B_* is slight whereas the central wavelength shifts distinctly. Figure 2(c) demonstrates the induction principle of strain along the uniform FBG. The experimental results are shown in Fig. 3(b), where the central wavelength of FBG is tuned with the range of >20 nm but the spectral profile changes slightly. The theoretical explanation can be achieved from Eqs. (3) and (5) (see METHODS). Obviously, the bandwidth- and wavelength-tunable operations can be realized independently by the vertical and horizontal translations, respectively.

### Experimental results of controlled flexible laser

**Figure 4:**
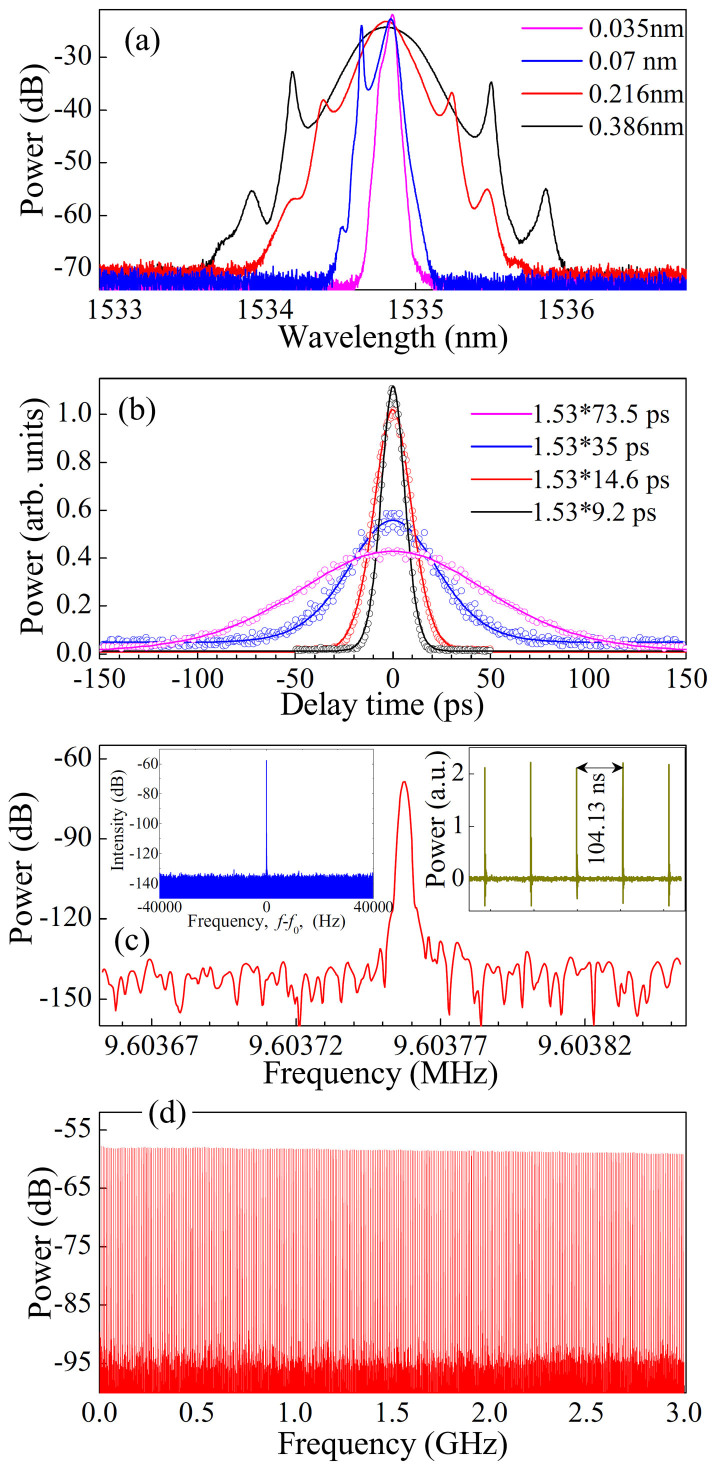
Typical laser characteristics. (a) Optical spectra at the FBG bandwidths (Δ*λ_B_*) of 0.17, 0.35, 0.71, and 1.48 nm (from inner to outer) by vertically translating the screw *G_z_* in Fig. 1(b). (b) Autocorrelation traces of the experimental data (circle symbols) and sech^2^–shaped fit (solid curves). The FWHM spectral bandwidths (Δ*λ*) and the corresponding pulse widths (Δ*τ*) are about 0.035 nm and 73.5 ps, 0.07 nm and 35 ps, 0.216 nm and 14.6 ps, and 0.386 nm and 9.2 ps, respectively. (c) Fundamental RF spectrum with the resolution of 2 Hz and the span of 200 Hz. Inset in the top left corner: RF spectrum with the resolution of 10 Hz and the span of 80 kHz. Inset in the top right corner: oscilloscope traces with the separation of ~104.13 ns, corresponding to 9.60376 MHz of the fundamental harmonic frequency that is independent of the pump power. (d) Wideband RF spectrum up to 3 GHz.

Self-starting mode-locking operation starts at the pump power of ~19 mW. With the appropriate setting of the polarization controller and the pump power, the proposed laser delivers the pulses with different widths by vertically adjusting the FBG (i.e., translating the screw *G_z_* along *z*-axis, as shown in Fig. 1(b)). The typical output spectra are shown in Fig. 4(a) with the central wavelength of ~1535 nm. The corresponding autocorrelation traces of the experimental data and the sech^2^–shaped fit are shown in Fig. 4(b). The full width at half maximum (FWHM) spectral bandwidths are about 0.035, 0.07, 0.216, and 0.386 nm at the FBG bandwidths of 0.17, 0.35, 0.71, and 1.48 nm, respectively. The corresponding pulse widths (Δ*τ*) are about 73.5, 35, 14.6, and 9.2 ps, respectively. Then, the calculated time-bandwidth products (TBPs) are about 0.33, 0.31, 0.40, and 0.45, respectively, which are close to the value of the transform-limited sech^2^-shaped pulses. The fluctuation of TBPs originates from the variations of the FBG bandwidth, the optical spectrum, and the total dispersion of laser cavity. The radio frequency (RF) spectra in Fig. 4(c) give a signal-to-noise ratio of >60 dB (>10^6^ contrast), showing low-amplitude fluctuations and good mode-locking stability^59^. No spectrum modulation is observed over 3 GHz in Fig. 4(d), indicating no Q-switching instability.

**Figure 5:**
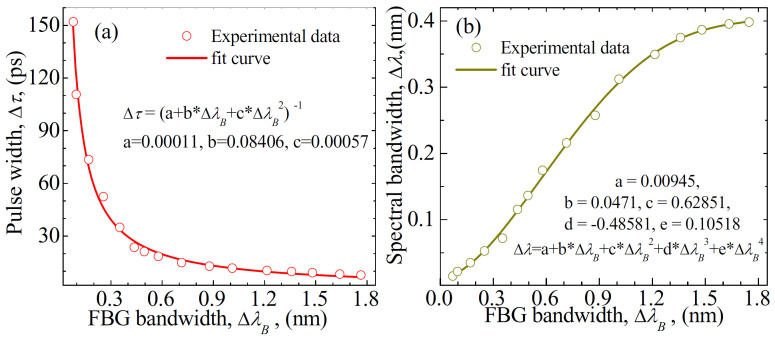
(a) Pulse width Δ*τ* and (b) spectral bandwidth Δ*λ* with respect to the FBG bandwidth Δ*λ_B_*. The beam is translated vertically, i.e., translating the screw *G_z_* along the direction of *z*-axis.

Figures 5(a) and 5(b) show the relationships of the pulse width Δ*τ* and the spectral bandwidth Δ*λ* with respect to the FBG bandwidth Δ*λ_B_*, respectively. The corresponding TBPs are ranging from ~0.31 to ~0.47 for the experimental data. The symbols and solid curves denote the typically experimental data and the fit curves, respectively. In the experiments, the maximum of pulse width Δ*τ* can be up to ~152 ps and the minimum of Δ*τ* can be down to ~7 ps. Note that the tuning range of Δ*τ* is limited by the FBGs. We can observe from Fig. 5(a) that the pulse width Δ*τ* approximately decreases with a rational function according to the FBG bandwidth Δ*λ_B_*. The rational equation that produces the best fit is as 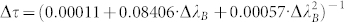. It is seen from Fig. 5(b) that the spectral bandwidth Δ*λ* approximately increases with a polynomial function of degree 5 with respect to Δ*λ_B_*. The polynomial equation that produces the best fit is as 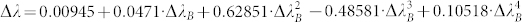. Figure 5(b) illustrates that the spectral bandwidth Δ*λ* of pulses approximately linearly increases along with Δ*λ_B_* for Δ*λ_B_* < 1 nm, whereas it changes slightly for Δ*λ_B_* > 1.5 nm. The origin of such behavior can be explained as follows. The different parts of FBG reflect the different wavelengths so that the round-trip distances for different frequencies of a pulse are different. When the spectral bandwidth of FBG is large enough, only a part of FBG spectra is employed in the mode-locking operation of fiber lasers and, then, the FBG with larger reflection bandwidth has a slight influence on the laser bandwidth.

**Figure 6:**
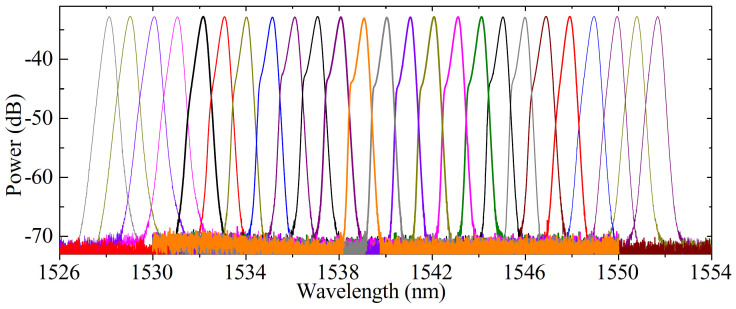
Output spectra of laser by horizontally translating the screw *G_x_* along the direction of *x*-axis.

Figure 6 demonstrates the typical output spectra by horizontally translating the screw *G_x_* along the direction of *x*-axis. The experimental results show that the spectral bandwidth Δ*λ* and pulse width Δ*τ* change slightly although the central wavelength of pulses is tuned evidently, indicating the stability of our output pulses. It is seen from Fig. 6 that the tuning range of wavelength is >20 nm with the FWHM spectral bandwidth of ~0.3 nm. The fluctuation of spectral profile originates from the dispersion variation of FBG when the central wavelength of FBG is tuned. The tuning range of wavelength is determined by the FBG. The experimental observations show that our laser can long-termed stably work for both width-tunable and wavelength-tunable operations. It attributes to the intrinsical merit of the CNT-based SA, which is insensitive to the environmental perturbations and the polarization of pulses.

## Discussion

In the experiments, the bandwidth of FBGs limits the tuning range of pulse width, as shown in Fig. 5(a). Theoretically, the proposed laser can deliver the pulses with the width of <1 ps if FBG in Fig. 1 is optimized. It is seen from Fig. 5(a) that the pulse width Δ*τ* of laser can be less than 1 ps when the FBG bandwidth Δ*λ_B_* is more than 11 nm. Although it is hard to fabricate a uniform FBG with the bandwidth of >2 nm, the bandwidth of the chirped FBGs can be up to 30 nm easily^54^. We can observe from Fig. 3(a) that the bandwidth Δ*λ_B_* of the uniform FBG can be extended up to 4 nm by vertically translating the screw *G_z_*, as shown in Fig. 1(b). However, the reflectivity of FBG decreases along with the increase of Δ*λ_B_*. For instance, the reflectivity of FBG is less than 30% for Δ*λ_B_* ≈ 2.5 nm. As a result, this laser will not operate when Δ*λ_B_* is extended to be more than 2.5 nm.

## Methods

### Principle of controlled operation

When the beam is bent or elongated (Fig. 2), linearly varying strain along its thickness or length is achieved. The strain response of FBG originates from both the physical elongation of the grating and the change in the refractive index due to photoelastic effects. The central wavelength variation of FBG, Δ*λ_c_*, induced by the strain *ε* along its axial direction is given by^56^,^57^
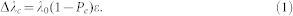
Here *λ*_0_ is the initial Bragg wavelength of the grating. *P_e_* is the effective photoelastic coefficient (approximately equal to 0.22), which is relative to the fiber Poisson ratio and the effective refractive index of the fiber core. 1-*P_e_* is the strain tuning coefficient. By vertically translating the screw *G_z_* along *z*-axis in Fig. 1(b), the strain introduced to the beam during bending is transferred to the FBG and induces an axial strain gradient along the grating, i.e.,
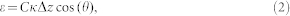
where *κ* is the curvature of the neutral layer of beam, Δ*z* is the vertical distance measured from the neutral layer, and *C* (0 < *C* < 1) is a constant introduced to describe the efficiency of strain transfer from the beam to the grating. The variation of Bragg wavelength is proportional to the local axial strain along the grating and the chirp of grating can be achieved when the beam is bent. The strain can be approximately proportional to *κ* and the grating length^57^,^58^. Then, the reflection bandwidth variation of FBG can be expressed as^57^
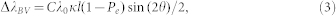
where *l* is the length of the grating.

By horizontally translating the screw *G_x_* along *x*-axis in Fig. 1(b), the strain introduced to the grating can be approximated by
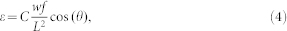
where *f* is the horizontal displacement at the end of beam. Substituting Eq. (4) into Eq. (1), we can achieve the central wavelength change of FBG when the translation is along the direction of *x*-axis, i.e.,
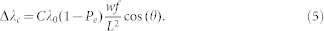
In this case, the reflection bandwidth variation of FBG can be approximated to zero, i.e., Δ*λ_BV_* ≈ 0. In fact, when the screw *G_x_* in Fig. 1(b) is translated horizontally, the angle between the axis of FBG and the vertical plane of translation is zero, i.e., *θ* = 0. Thus, Δ*λ_BV_* is equal to zero according to Eq. (3).

### Measurement method

An optical spectrum analyzer (Yokogawa AQ-6370), an autocorrelator (APE *Pulse*Check SM1600), a 6-GHz oscilloscope (LeCroy WaveMaster 8600A), a radio-frequency (RF) analyzer (Agilent E4447A), and a 10-GHz photodetector are used to measure the laser output performances.

## Author Contributions

X.L. proposed the laser system and wrote the manuscript text. Y.C. performed the main experimental results. All authors discussed the results and substantially contributed to the manuscript.

## References

[b1] Gabzdyl J. Fibre lasers make their mark. Nat. Photon. 2, 21–23 (2008).

[b2] Renninger W. H. & Wise F. W. Optical solitons in graded-index multimode fibres. Nat. Commun. 4, 1719 (2013). 23591886 10.1038/ncomms2739

[b3] EI-Taher A., Kotlicki O., Harper P., Turitsyn S. & Scheuer J. Secure key distribution over a 500 km long link using a Raman ultra-long fiber laser. Laser Photon. Rev. 8, 436–442 (2014).

[b4] Turitsyna E. G. *et al.* The laminar-turbulent transition in a fibre laser. Nat. Photon. 7, 783–786 (2013).

[b5] Freudiger C. W. *et al.* Stimulated Raman scattering microscopy with a robust fibre laser source. Nat. Photon. 8, 153–159 (2014). 10.1038/nphoton.2013.360 PMC4193905 25313312

[b6] Turitsyn S. K. *et al.* Random distributed feedback fibre laser. Nat. Photon. 4, 231–235 (2010).

[b7] Brida D., Krauss G., Sell A. & Leitenstorfer A. Ultrabroadband Er: fiber lasers. Laser Photon. Rev. 8, 409–428 (2014).

[b8] Jauregui C., Limpert J. & Tünnermann A. High-power fibre lasers. Nat. Photon. 7, 861–867 (2013).

[b9] Peccianti M. *et al.* Demonstration of a stable ultrafast laser based on a nonlinear microcavity. Nat. Commun. 3, 765 (2012). 22473009 10.1038/ncomms1762 PMC3337978

[b10] Limpert J. *et al.* Ultrafast fiber lasers for strong-field physics experiments. Laser Photon. Rev. 5, 634–646 (2011).

[b11] Xu C. & Wise F. W. Recent advances in fibre lasers for nonlinear microscopy. Nat. Photon. 7, 875–882 (2013). 10.1038/nphoton.2013.284 PMC3887125 24416074

[b12] Jackson S. D. Towards high-power mid-infrared emission from a fibre laser. Nat. Photon. 6, 423–431 (2012).

[b13] Grelu P. & Akhmediev N. Dissipative solitons for mode-locked lasers. Nat. Photon. 6, 84–92 (2012).

[b14] Dudley J. M., Finot C., Richardson D. J. & Millot G. Self-similarity in ultrafast nonlinear optics. Nat. Phys. 3, 597–603 (2007).

[b15] Eigenwillig C. M. *et al.* Picosecond pulses from wavelength-swept continuous-wave Fourier domain mode-locked lasers. Nat. Commun. 4, 1848 (2013). 23673633 10.1038/ncomms2870

[b16] Tsatourian V. *et al.* Polarisation dynamics of vector soliton molecules in mode locked fibre laser. Sci. Rep. 3, 3154 (2013). 24193374 10.1038/srep03154 PMC3818655

[b17] Fermann M. E. & Hartl I. Ultrafast fibre lasers. Nat. Photon. 7, 868–874 (2013).

[b18] Barbieri S. *et al.* Phase-locking of a 2.7-THz quantum cascade laser to a mode-locked erbium-doped fibre laser. Nat. Photon. 4, 636–640 (2010).

[b19] Oktem B., Ulgudur C. & Ilday F. Soliton-similariton fibre laser. Nat. Photon. 4, 307–311 (2010).

[b20] Leonetti M., Conti C. & Lopez C. The mode-locking transition of random lasers. Nat. Photon. 5, 615–617 (2011).

[b21] Quarterman A. H. *et al.* A passively mode-locked external-cavity semiconductor laser emitting 60-fs pulses. Nat. Photon. 3, 729–731 (2009).

[b22] Rafailov E. U., Cataluna M. A. & Sibbett W. Mode-locked quantum-dot lasers. Nat. Photon. 1, 395–401 (2007).

[b23] Jones D. J. *et al.* Carrier-envelope phase control of femtosecond mode-locked lasers and direct optical frequency synthesis. Science 288, 635–639 (2000). 10784441 10.1126/science.288.5466.635

[b24] Liu X. Hysteresis phenomena and multipulse formation of a dissipative system in a passively mode-lockedfiber laser. Phys. Rev. A 81, 023811 (2010).

[b25] Matsas V. J., Newson T. P., Richardson D. J. & Payne D. N. Self-starting, passively mode-locked fibre ring soliton laser exploiting non-linear polarization rotation. Electron. Lett. 28, 1391–1393 (1992).

[b26] Wise F. W., Chong A. & Renninger W. H. High-energy femtosecond fiber lasers based on pulse propagation at normal dispersion. Laser Photon. Rev. 2, 58–73 (2008).

[b27] Tang D. Y., Zhang H., Zhao L. M. & Wu X. Observation of high-order polarization-locked vector solitons in a fiber laser. Phys. Rev. Lett. 101, 153904 (2008). 18999601 10.1103/PhysRevLett.101.153904

[b28] Liu X. M. & Mao D. Compact all-fiber high-energy fiber laser with sub-300-fs duration. Opt. Express 18, 8847–8852 (2010). 20588729 10.1364/OE.18.008847

[b29] Nelson L. E., Jones D. J., Tamura K., Haus H. A. & Ippen E. P. Ultrashort-pulse fiber ring lasers. Appl. Phys. B 65, 277–294 (1997).

[b30] Wang L. *et al.* Observations of four types of pulses in a fiber laser with large net-normal dispersion. Opt. Express 19, 7616–7624 (2011). 21503070 10.1364/OE.19.007616

[b31] Yun L., Liu X. M. & Mao D. Observation of dual-wavelength dissipative solitons in a figure-eight erbium-doped fiber laser. Opt. Express 20, 20992–20997 (2012). 23037222 10.1364/OE.20.020992

[b32] Doran N. J. & Wood D. Nonlinear-optical loop mirror. Opt. Lett. 13, 56–58 (1988). 19741979 10.1364/ol.13.000056

[b33] Salhi M., Haboucha A., Leblond H. & Sanchez F. Theoretical study of figure-eight all-fiber laser. Phys. Rev. A 77, 033828 (2008).

[b34] Zhang Z. Y. *et al.* 1.55 mm InAs/GaAs quantum dots and high repetition rate quantum dot SESAM mode-locked laser. Sci. Rep. 2, 477 (2012). 22745898 10.1038/srep00477 PMC3384963

[b35] Keller U. Recent developments in compact ultrafast lasers. Nature 424, 831–838 (2003). 12917697 10.1038/nature01938

[b36] Kornaszewski L. *et al.* SESAM-free mode-locked semiconductor disk laser. Laser Photon. Rev. 6, L20–L23 (2012).

[b37] Mao D., Liu X. & Lu H. Observation of pulse trapping in a near-zero dispersion regime. Opt. Lett. 37, 2619–2621 (2012). 22743473 10.1364/OL.37.002619

[b38] Akhmediev N. N., Soto-Crespo J. M., Cundiff S. T., Collings B. C. & Knox W. H. Phase locking and periodic evolution of solitons in passively mode-locked fiber lasers with a semiconductor saturable absorber. Opt. Lett. 23, 852–854 (1998). 18087363 10.1364/ol.23.000852

[b39] Bao Q. *et al.* Monolayer graphene as a saturable absorber in a mode-locked laser. Nano Res. 4, 297–307 (2011).

[b40] Martinez A. & Sun Z. Nanotube and graphene saturable absorbers for fibre lasers. Nat. Photon. 7, 842–845 (2013).

[b41] Sobon G., Sotor J. & Abramski K. M. Passive harmonic mode-locking in Er-doped fiber laser based on graphene saturable absorber with repetition rates scalable to 2.22 GHz. Appl. Phys. Lett. 100, 161109 (2012).

[b42] Cui Y. & Liu X. Graphene and nanotube mode-locked fiber laser emitting dissipative and conventional solitons. Opt. Express 21, 18969–18974 (2013). 23938811 10.1364/OE.21.018969

[b43] Ma J. *et al.* Wavelength-versatile graphene-gold film saturable absorber mirror for ultra-broadband mode-locking of bulk lasers. Sci. Rep. 4, 5016 (2014). 24853072 10.1038/srep05016 PMC4031483

[b44] Solodyankin M. A. *et al.* Mode-locked 1.93 μm thulium fiber laser with a carbon nanotube absorber. Opt. Lett. 33, 1336–1338 (2008). 18552950 10.1364/ol.33.001336

[b45] Liu X. *et al.* Versatile multi-wavelength ultrafast fiber laser mode-locked by carbon nanotubes. Sci. Rep. 3, 2718 (2013). 24056500 10.1038/srep02718 PMC3779847

[b46] Wang F. *et al.* Wideband-tuneable, nanotube mode-locked, fibre laser. Nat. Nanotechnol. 3, 738–742 (2008). 19057594 10.1038/nnano.2008.312

[b47] Xu X. T. *et al.* Passively mode-locking erbium-doped fiber lasers with 0.3 nm single-walled carbon nanotubes. Sci. Rep. 4, 6761 (2014). 25342292 10.1038/srep06761 PMC4208059

[b48] Set S. Y., Yaguchi H., Tanaka Y. & Jablonski M. Ultrafast fiber pulsed lasers incorporating carbon nanotubes. IEEE J. Sel. Top. Quant. Electron. 10, 137–146 (2004).

[b49] Nozaki Y., Nishizawa N., Omoda E., Kataura H. & Sakakibara Y. Power scaling of dispersion-managed Er-doped ultrashort pulse fiber laser with single wall carbon nanotubes. Opt. Lett. 37, 5079–5081 (2012). 23258011 10.1364/OL.37.005079

[b50] Kurashima Y., Yokota Y., Miyamoto I., Kataura H. & Sakakibara Y. Mode-locking nanoporous alumina membrane embedded with carbon nanotube saturable absorber. Appl. Phys. Lett. 94, 223102 (2009).

[b51] Han D. D. *et al.* Simultaneous picosecond and femtosecond solitons delivered from a nanotube-mode-locked all-fiber laser. Opt. Lett. 39, 1565–1568 (2014). 24690839 10.1364/OL.39.001565

[b52] Chong A., Renninger W. H. & Wise F. W. Properties of normal-dispersion femtosecond fiber lasers. J. Opt. Soc. Am. B 25, 140–148 (2008).

[b53] Sun Z. *et al.* A Stable, Wideband Tunable, Near Transform-Limited, Graphene Mode-Locked, Ultrafast Laser. Nano Res. 3, 653–660 (2010). 10.1021/nn901703e 20099874

[b54] Kashyap R. Fiber Bragg Gratings. Academic Press, San Diego (1999).

[b55] Canning J. Fibre gratings and devices for sensors and lasers. Laser Photon. Rev. 2, 275–289 (2008).

[b56] Kersey A. D. Fiber Grating Sensors. J. Lightwave Technol. 15, 1442–1463 (1997).

[b57] Dong X. Y., Guan B. O., Yuan S. Z., Dong X. Y. & Tam H. Strain gradient chirp of uniform fiber Bragg grating without shift of central Bragg wavelength. Opt. Commun. 202, 91–95 (2002).

[b58] Han Y. & Lee S. Tunable dispersion compensator based on uniform fiber Bragg grating and its application to tunable pulse repetition-rate multiplication. Opt. Express 13, 9224–9229 (2005). 19503122 10.1364/opex.13.009224

[b59] Von der Linde D. Characterization of the noise in continuously operating mode-locked lasers. Appl. Phys. B 39, 201–217 (1986).

